# Pulsed Field Ablation: A Review of Preclinical and Clinical Studies

**DOI:** 10.3390/bioengineering12040329

**Published:** 2025-03-22

**Authors:** Andrew P. Sullivan, Martin Aguilar, Zachary Laksman

**Affiliations:** 1 Department of Medicine, University of British Columbia, Vancouver, BC V5Z 1M9, Canada; apsullivan@mun.ca; 2 Montreal Heart Institute, Department of Medicine, Université de Montréal, Montréal, QC H1T 1C8, Canada; martin.aguilar@umontreal.ca

**Keywords:** irreversible electroporation, pulsed field ablation, atrial fibrillation

## Abstract

Pulsed field ablation (PFA) is an emerging technology that utilizes ultra-short high-voltage electric pulses to create nanopores in cell membranes, leading to cell death through irreversible electroporation (IRE). PFA is touted to be highly tissue-selective, which may mitigate the risk of collateral injury to vital adjacent structures. In the field of cardiac electrophysiology, initial studies have shown promising results for acute pulmonary vein isolation (PVI) and lesion durability, with overall freedom from recurrent atrial arrhythmia comparable to traditional thermal ablation modalities. While further large studies are required for long-term efficacy and safety data, PFA has the potential to become a preferred energy source for cardiac ablation for some indications. This review outlines the basic principles and biophysics of IRE and its application to cardiac electrophysiology through a review of the existing preclinical and clinical data.

## 1. Introduction

Cardiac ablation is an effective treatment strategy for a wide range of arrhythmias. The goal of ablation is to alter the electrophysiologic properties of arrhythmogenic substrate by creating permanent lesions within the myocardium and thus interrupting the propagation of abnormal electrical impulses. Lesion formation traditionally requires the delivery of an energy source facilitated by a catheter. The ideal energy source can accomplish permanent transmural ablation of the intended tissue while sparing adjacent structures. Energy sources have evolved to optimize both efficacy and safety.

The initial modality used for ablation was direct current energy, which involved high-energy shocks up to 500 J delivered from a conventional transvenous pacing catheter [1,2]. While effective in achieving up-front atrioventricular nodal conduction block, lesion formation was inconsistent, and thus, patients developed recurrent arrhythmia. Furthermore, the large energy delivery to a small, targeted area was associated with complications of arcing, barotrauma, and proarrhythmic side effects.

Radiofrequency (RF) energy ablation was subsequently developed and remains a first-line energy source for ablation. RF employs high-frequency (500–750 kHz) alternating current, causing ions within the tissue to oscillate, leading to frictional heating [3]. The temperature at the electrode increases rapidly to greater than 50 °C, creating a thermal gradient and resulting in coagulative necrosis in the cells due to the denaturation of proteins and the destruction of cellular structures [4]. Heat conduction is transmitted to adjacent structures via principles of thermal diffusion [5]. The catheter tip temperature is influenced by electrode–tissue contact, the location of the temperature electrode, and convective cooling provided by the blood tissue interface, as well as catheter-related irrigation [6]. The thermal conductivity of the tissue influences the spread of heat from the ablation zone to deeper surrounding structures. This is relevant given multiple vital collateral structures, including the esophagus, phrenic nerve, and coronary arteries.

An alternative and widely employed ablation energy source is cryothermal ablation. Lesion formation is achieved through cryothermy at the catheter–tissue interface. A pressurized cryo-refrigerant is delivered to the catheter, resulting in a temperature drop to below −40 °C, triggering intracellular ice crystal formation. This results in an extracellular osmotic gradient, leading to cell shrinkage and reduction in cellular pH, causing protein dysfunction and irreversible mitochondrial damage [7]. Subsequent microvascular hemorrhage and inflammation ensue, followed by replacement fibrosis [8]. Similar to RF, cryoablation is also associated with damage to surrounding structures, resulting in risk of complications including phrenic nerve palsy and pulmonary vein (PV) stenosis.

Stereotactic body radiation therapy (SBRT) is an emerging non-invasive ablation technique that employs focused high-energy external radiation to precisely target and modify arrhythmogenic substrate without the need for intra-cardiac catheters. As such, it eliminates the need for invasive procedures, which is particularly beneficial to patients with complex arrhythmias or in those whom conventional approaches have failed. Most of the evidence for SBRT is in patients with refractory ventricular tachycardia (VT); however, it has also been studied in atrial fibrillation (AF) [9,10]. Electroanatomic mapping can be performed using a surface ECG electrode vest that is then integrated with multi-modality imaging, including a combination of cardiac magnetic resonance imaging (MRI), cardiac computed tomography (CT), and cardiac single-photon emission CT (SPECT). This is followed by radiation treatment planning and delivery in a single fraction of 25 to 35 Gy. The mechanism of action of cardiac SBRT is not fully understood. It is hypothesized that ionizing radiation induces double-stranded DNA breaks triggering programmed cell death and subsequently dense transmural fibrosis, which decreases propensity for re-entry [11].

Pulsed field ablation (PFA) has emerged as a promising novel ablation modality that utilizes irreversible electroporation (IRE) to create ablation lesions quickly with an energy source that is primarily non-thermal while sparing adjacent structures. This modality improves the safety of ablation by reducing the risk of serious complications.

## 2. Electroporation Biophysics

Pulsed field ablation (PFA) is a novel ablation technology that uses IRE to create ablation lesions by inducing cell death through a non-thermal approach. Each cell possesses a resting transmembrane voltage (TMV), which is essential for functions like proliferation, migration, and maintaining cell shape [12]. In eukaryotic cells, TMV typically ranges between −40 and −70 mV and is sustained by membrane ion channels and pumps, resulting in a negatively charged interior relative to the exterior [13]. When a cell is exposed to electric pulses, an electrical field is formed. The electrical field causes an induced TMV that is sustained for the duration of the exposure and is proportional to the strength of the electric field [13]. When high-voltage pulses of short duration are applied, the phenomenon of electroporation results in increased permeability of the cell membrane via formation of pores, lipid oxidation, and protein damage [14,15]. Membrane electropermeabilization allows for the entry of membrane-impermeant molecules into the cell and outflow of intracellular biomolecules. This phenomenon occurs in under a microsecond after the application of the electric pulse, provided the induced TMV exceeds a critical electroporation threshold [13]. The threshold of electric field required to induce electroporation is different for each cell type and is dependent on many other variables, including cell size, local membrane curvature, temperature, and osmotic pressure [13,16]. There exists a higher critical value at which point cell damage becomes irreversible (IRE) and cell death occurs [17,18]. If this threshold is not exceeded, cellular homeostasis and plasma membrane integrity can be re-established through a process of resealing once the electric field is removed [19].

**Figure 1:**
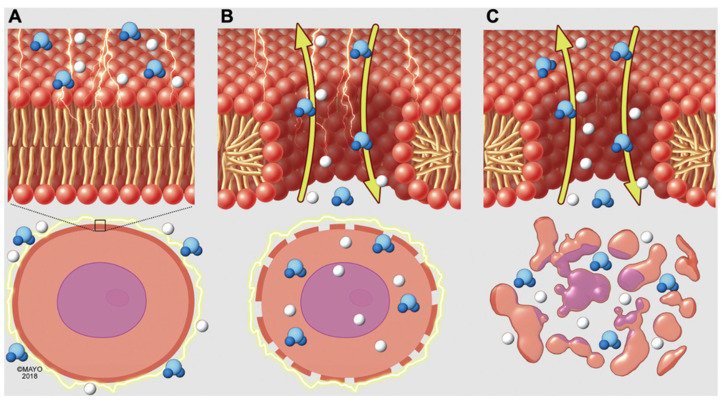
Schematic illustration of the cellular mechanism of irreversible electroporation (Maor 2019 [20], reproduced with permission). Electric field-induced nanopore formation in cell membrane showing progression to cell death (**A**–**C**).

If the TMV threshold is exceeded, the aqueous lipid bilayer pores fail to seal and a cascade of intracellular processes ensue resulting in cell death (Figure 1) [20]. Multiple variables influence a cell’s ability to reseal, including temperature, hydraulic stress, cellular cytoskeleton characteristics, membrane fluidity, and lipid peroxidation [21,22,23,24,25]. Cell death due to electroporation is not fully understood; however, multiple mechanisms may occur, including apoptosis, necrosis, necroptosis, and pyroptosis [14,26]. One proposed mechanism of cell death is due to calcium influx from the extracellular space, which triggers disassembly of cortical cytoskeleton and adenosine triphosphate (ATP) depletion. At the same, danger-associated molecular pattern (DAMP) molecules leak out of the cell triggering causing immune activation. Reactive oxygen species are also generated, further destabilizing cell membranes and leading to cell death [14,27].

Understanding the electroporation phenomenon requires calculating the induced TMV. For simple cell structures, like spherical cells, this can be analytically derived using the Schwan equation: Δ*M* = 1.5*ER*cosθ, where ΔM represents the induced TMV, *E* is the external electric field, *R* is the cell radius, and θ is the angle relative to the field direction. This equation shows that the induced TMV is directly proportional to both the applied electric field and the cell radius, meaning larger cells experience higher TMV and, thus, electroporation at lower field intensities [28]. However, this model assumes cells are spherical and isolated, which is rarely true in biological tissues. Irregularly shaped or densely packed cells require more complex methods for accurate TMV calculation. Since cardiac cells are not spherical, these limitations highlight the need for more advanced models to predict electroporation in complex tissues accurately. The greatest induced TMV occurs when the electric field is perpendicular to the membrane, particularly on the sides facing the electrodes, with the TMV being higher on the anode side due to the additional contribution of the resting TMV. Anodal electroporation, therefore, can occur at lower field strengths compared to cathodal electroporation [29].

**Figure 2:**
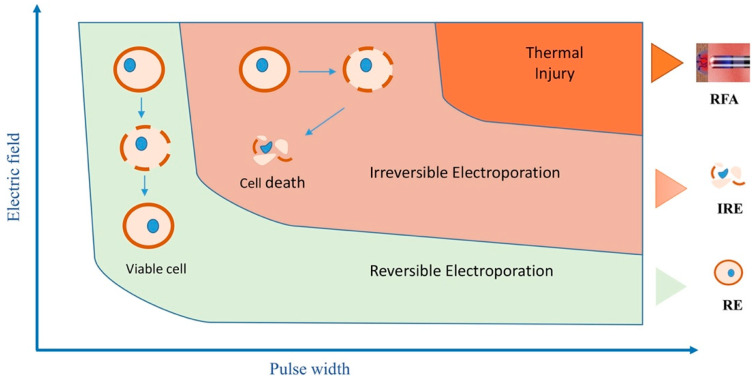
Relationship between electrical pulse and electric field strength (Tabaja 2023 [16], reproduced with permission). At greater voltages and longer pulses, the risk of progression from IRE to thermal damage increases.

Mitigating the possibility of thermal damage is an important consideration in IRE. When voltage reaches a sufficiently high level, electroporation triggers tissue heating, following Joule’s first law: Q = I^2^ R t, where Q is heat, R is resistance, I is current, and t is duration of current application. To avoid this, energy is administered in ultra-rapid pulse durations ranging from microseconds to nanoseconds. Figure 2 illustrates this concept that with longer duration of pulse width at high electric field strength, the risk for heating and therefore thermal injury increases. This approach effectively disperses any generated heat through the cooling mechanisms of conduction, convection, and the presence of surrounding blood flow. While some studies note minor heating effects, others show no temperature rise, even at high voltages [30,31]. Methods to enhance IRE-induced apoptosis without increasing electric field strength include using repetitive pulses, optimizing pulse duration and shape, altering medium conductivity, and introducing calcium, dramatically decreasing cell ATP levels [32,33,34].

## 3. Pulsed Field Ablation Technology

**Figure 3:**
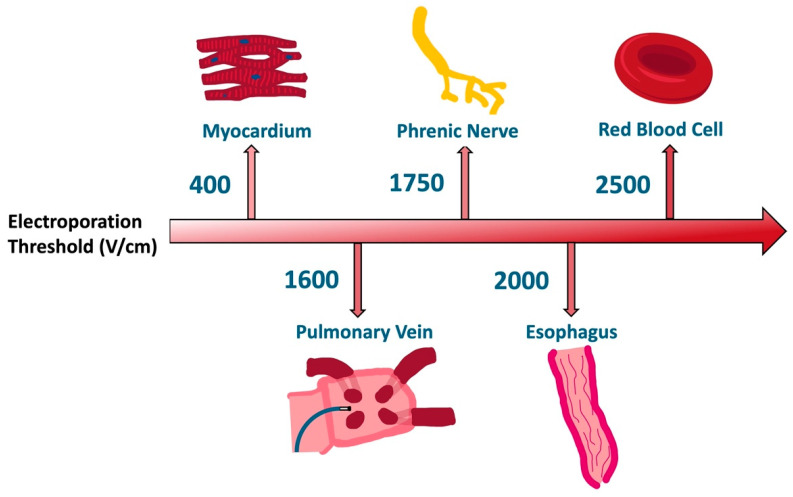
Representative transmembrane voltage thresholds for irreversible electroporation of organs in proximity to atrial tissue [16,35,40,41,42]. The data for tissue-specific electroporation thresholds come from a variety of in vitro and in vivo preclinical experimental setups and models and thus numerical values presented are not absolute or directly comparable.

The goal of IRE is to deliver high energy for effective target tissue ablation while avoiding thermal injury or damage to neighboring structures. Each tissue type has a unique electroporation threshold that is based on cellular properties such as capacitance, dipole potential, and lipid hydrophobic tail characteristics that influence the lipid bilayer structure [16]. The data for tissue specific electroporation thresholds come from a variety of in vitro and in vivo preclinical experimental setups and models [16,35,36,37,38,39,40,41,42]. Thus, the specific threshold values are not absolute and may change with different pulsed electric field protocols and delivery platforms. As shown in Figure 3, it is suggested that atrial cardiomyocytes have a lower electroporation threshold than adjacent structures including the esophagus, phrenic nerve, pulmonary veins, and red blood cells [35]. In addition to voltage, pulse duration is important in achieving optimal energy delivery. Longer pulse duration requires lower voltages to elicit the same number of electroporated cells in a nonlinear relationship [28,43]. The number of pulses administered may also be important in modulating IRE; however, equipoise still exists in this regard [44].

**Figure 4:**
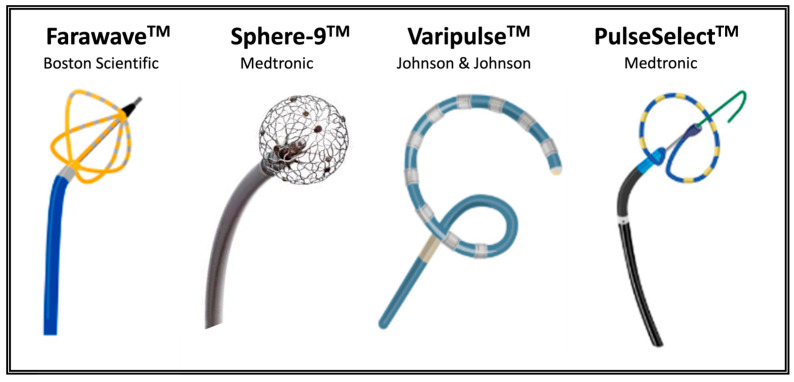
Schematic illustration of currently approved catheters used for pulsed field ablation (Iyengar 2023 [46]/CC BY 4.0, reproduced and modified with permission). Significant variations in catheter design and electrode configurations exist, all with the goal of creating effective ablation lesions via irreversible electroporation.

The catheter is an essential aspect of almost any cardiac ablation system and is responsible for transmitting the energy source to a highly targeted region of interest within the heart. There are multiple different catheter designs for delivering IRE that have been studied in pre-clinical and clinical trials (Figure 4). They exist in various shapes, such as balloons, baskets, and circular and linear configurations. The lesion produced is a consequence of both the pulse recipe and the catheter design. The design affects not only the field distribution but also less readily simulated variables such as the likelihood of getting close tissue proximity to enable effective ablations. Additionally, cell orientation affects electroporation relative to the direction of the electric field, with electroporation being more effective when the electric field is perpendicular to the cell membrane rather than parallel. Therefore, a nonlinear catheter design combined with rotational movement may enhance the effect on cells by compensating for variable cell alignment [16,45].

## 4. Preclinical Studies

The aim of this review is not to include an exhaustive description of every preclinical study but to provide a narrative overview of how animal studies have bridged the gap between IRE biophysics and clinical medicine as it pertains to safety and efficacy outcomes in PFA. Lavee et al. conducted the first in vivo testing of IRE in 2007 using a clamp containing two parallel electrodes to the atrial epicardium of five pigs [47]. Histologic evaluation of the excised hearts 24 h post-ablation revealed complete transmural destruction at the site of electrode application in all lesions. Hong et al. showed similar lesion formation efficacy in four sheep and demonstrated only a minimal inflammatory reaction at the ablation site [30]. This was followed up with work by Van Driel et al. demonstrating the safety of IRE in preventing pulmonary vein injury or stenosis [48]. Koruth et al. showed that biphasic pulse delivery reduced skeletal muscle engagement and higher PV isolation durability than monophasic [49]. The safety of IRE compared to RF in preventing esophageal injury was shown by Yavin et al. by direct ablation inside the esophagus [50]. Hsu et al. demonstrated that no collateral damage to adjacent structures occurred even with supratherapeutic IRE energy of 1800 V [51]. Song et al. showed no histological evidence of esophageal energy 16 weeks following IRE at 2000 V/cm directly to the esophagus in the rabbit model, after regeneration and repair [40].

Most of the research in the preclinical and clinical domains for IRE pertains to the left atrium, given its relevance to pulmonary vein isolation in the ablation of AF. Given the significant structural differences between atrial and ventricular myocardium, dedicated studies of ventricular ablation are essential to understanding whether IRE could effectively treat ventricular arrhythmias. Wittkampf et al. were the first to explore the use of IRE on left ventricular (LV) tissue by performing LV epicardial ablation in porcine models using a variety of energy levels from 50 to 200 J. Histologic analysis was performed 3 weeks following ablation and demonstrated a correlation between lesion depth and current strength used. A follow-up study showed that epicardial IRE ablation could create transmural ventricular lesions. Transient coronary artery spasm was observed; however, no coronary artery narrowing was noted at the 3-month follow-up [52]. A limitation of this study is that energy is reported in joules, which is not comparable to voltage reported in other studies. The important take-away, however, is the relationship between energy delivery and lesion depth and the ability for PFA to produce deep ventricular lesions. Livia et al. investigated the role of IRE in the ablation of Purkinje fibers in a canine model for the indication of ventricular fibrillation ablation. Purkinje potentials were consistently eradicated with IRE at voltages between 750–2500 V [53]. In a swine infarct model, PFA showed effective ablation of surviving islands of myocardium within and around infarcted LV substrate and produced significantly deeper lesions compared to RF [54]. Younis et al. confirmed these findings and demonstrated that PFA achieved transmural ventricular lesions, whereas RF was restricted to the endocardial layer and unable to consistently penetrate deep within scar [55].

## 5. Clinical Studies

A non-exhaustive list of clinical studies of PFA is summarized chronologically in Table 1. Studies were selected for inclusion based on a semi-systematic search through PubMed database employing key-words “pulsed field ablation” and “electroporation”. Studies identified from previous reviews and publications were also included.

The first-in-human data of PFA for the treatment of AF were published in 2018 under the direction of Dr. Vivek Reddy [56]. A total of 22 patients from two centers in Europe underwent either percutaneously delivered endocardial or surgical epicardial ablation using monophasic waveform. Catheter ablation was 100% successful in obtaining PV isolation, whereas epicardial ablation had an 86% success rate with surgical box lesions. No acute adverse events were observed; however, no follow-up safety or efficacy data were reported.

In 2020, Reddy et al. reported on a hybrid lattice-tip ablation catheter (Sphere-9, Affera, Inc., Watertown, MA, USA) capable of toggling between RF and PFA delivery. The generator (HexaGen and Hexapulse, Affera, Inc., Newton, MA, USA) contains a dual design enabling switching between the two energy sources. They conducted a three-center, single-arm, first-in-human trial enrolling 76 patients with either paroxysmal or persistent AF. Forty patients underwent combined RF/PFA, and 36 had PFA alone. PFA was used for posterior LA due to proximity to the esophagus. In the patients with persistent AF, additional lesion sets of LA roof or posterior mitral isthmus were permitted. Acute PVI was achieved in 100% of cases with no device-related complications [57].

The clinical endpoint of freedom from AF recurrence was addressed in three clinical trials with similar designs: IMPULSE (A Safety and Feasibility Study of the IOWA Approach Endocardial Ablation System to Treat Atrial Fibrillation), PEFCAT (A Safety and Feasibility Study of the FARAPULSE Endocardial Ablation System to Treat Paroxysmal Atrial Fibrillation), and PEFCAT II (Expanded Safety and Feasibility Study of the FARAPULSE Endocardial Multi Ablation System to Treat Paroxysmal Atrial Fibrillation). These three studies were prospective, non-randomized, single-arm safety and feasibility trials that included a combined 121 patients with symptomatic paroxysmal AF. IMPULSE and PEFCAT used an over-the-wire single-shot pentaspline multielectrode PFA catheter (Farawave, Farapulse Inc., Menlo Park, CA, USA) capable of both “basket” and “flower” configurations. PEFCAT II used the same catheter for PVI but also included a deflectable focal PFA catheter (Faraflex, Farapulse Inc., Menlo Park, CA, USA) for investigation of the first-in-human treatment of cavotricuspid isthmus (CTI)-dependent atrial flutter. In the combined 121 patients, acute PVI was achieved in 100% of PVs. One hundred and ten patients underwent re-mapping in 3 months, demonstrating durable PVI in 84.1% of patients and 96% in those receiving the optimized biphasic energy PFA waveform. Fifteen patients were treated with a monophasic protocol that necessitated general anesthesia and neuromuscular paralysis in minimize muscle contraction. Compared to bipolar, there was significantly reduced PV durability at 45% of PV at time of remapping. Patients were monitored for one year through clinical visits, trans-telephonic monitoring, and 24 h Holter recordings to detect AF recurrence. After one year, the Kaplan–Meier estimate for freedom from AF was 81.1 ± 3.8%. Among the 49 patients treated with the optimized biphasic protocol, this estimate was 84.6 ± 6%. The primary safety event rate was low at 2.5% due to two instances of cardiac tamponade. No esophageal injury, phrenic nerve damage, or brain lesions were observed post-ablation [59]. Long-term data up to 5 years (median postprocedural follow-up 49 months) post-ablation showed that 73% of patients remained free from atrial arrhythmias and 68% free from AF/atrial flutter (AFL) and off Class I/III antiarrhythmic drugs. Excluding the 20 patients with recurrence in the first year, 89% remained free from late-onset recurrence for up to 5 years [74].

Loh et al. conducted a single-center, nonrandomized, prospective cohort study of 10 patients with symptomatic paroxysmal or persistent AF using a custom non-deflectable 8F, 14-polar IRE catheter with a variable hoop diameter between 16 and 27 mm. Acute bidirectional electrical PVI was achieved safely by single-pulse IRE ablation in all 10 patients [60]. The PersAFOne trial was a single-arm study evaluating biphasic, bipolar PFA using a multispline catheter (Farawave, Farapulse Inc., Menlo Park, CA, USA) for PVI and left atrial posterior wall (LAPW) ablation in patients with persistent AF. Twenty-five patients were enrolled with acute PVI achieved in 100% of cases with a mean ablation time of 22 min. LAPW was successful in 24/24 cases. CTI PFA was concurrently carried out in 13 patients using a focal tetraspline deflectable catheter (Faraflex, Farapulse Inc., Menlo Park, CA, USA) and was acutely successful in all cases. Invasive remapping was performed on average 82 days post-ablation, showing 96% PVI and 100% LAPW durability. There were no IRE-related adverse events [61].

PULSED AF was a prospective, global, multicenter, nonrandomized trial evaluating a circular-lasso-type nine-electrode catheter (PulseSelect, Medtronic, Inc., Minneapolis, MN, USA) for the treatment of paroxysmal and persistent AF. Three hundred patients were split evenly into 150 paroxysmal and 150 persistent. The primary combined endpoint of freedom from acute procedural failure, arrhythmia recurrence, or antiarrhythmic escalation though 12 months occurred in 66.2% of patients with paroxysmal AF and 55.1% with persistent AF. There were two serious procedure- and device-related adverse events (one cerebrovascular accident and one pericardial effusion requiring drainage) [62].

Following the approval of the first PFA system (Farawave, Farapulse, Menlo Park, CA, USA) in 2021, the MANIFEST-PF registry was created, encompassing 1568 consecutive patients from 24 European sites. PVI was achieved in 99.2%, with freedom from AF/AFL/AT in 78% (paroxysmal 81.6% and persistent 71.5%). The rate of major adverse events was low (1.9%), with no esophageal complications or symptomatic PV stenosis reported [63].

In 2023, Reddy et al. published a first-in-human study of a focal 9 mm lattice-tip catheter with the ability to toggle between PFA and RF. This single-arm study included 178 patients with paroxysmal or persistent AF. All patients received PFA for posterior LA ablation and half underwent either PFA or RF ablation for the anterior LA. Additional lesion sets including mitral isthmus line, LA roof line, and CTI were delivered at the operator’s discretion. All lesion sets (100%) were acutely successful. At 1 year, freedom from atrial arrhythmias was 78.3% (paroxysmal) and 77.9% (persistent). A single adverse event of inflammatory pericardial effusion not requiring intervention occurred [64].

The inspIRE study evaluated the safety and efficacy of a biphasic PFA system with a variable-loop circular catheter (Varipulse, Biosense Webster, Inc., Irvine, CA, USA) for treating paroxysmal AF. Two hundred and twenty-six patients from 13 centers across Europe and Canada were enrolled. Twelve-month freedom from atrial arrhythmia recurrence was 78.9%. Silent cerebral lesions were detected by brain MRI in 8 of 39 patients. All cerebral lesions were asymptomatic and resolved within 3 months of follow-up [65]. The Varipulse catheter was further studied in the admIRE trial at 33 centers, enrolling 277 patients with symptomatic drug-refractory paroxysmal AF. At 12 months, freedom from AF/AFL/AT and clinical success were 76.2% and 94.4%, respectively [66].

PFA was compared to cryoballoon (CB) ablation in a retrospective study of 400 patients with paroxysmal or persistent AF by Urbanek et al. Acute PVI was achieved in 100% of PFA and 98% of CB patients. Median procedure time was significantly shorter with PFA (34.5 min) compared to CB (50 min). Freedom from atrial arrhythmias at 1 year was 74.5% with PFA compared to 78.1% with CB. Persistent phrenic nerve palsy occurred in three patients with CB [67]. PFA was also compared to CB by Schipper et al. in a retrospective study of 108 patients. Acute PVI was achieved in 100% and 99.5% of PVs in PFA and CB, respectively. Arrhythmia-free survival after 273 ± 129 days was 74% in PFA and 72% in CB. There were two complications with PFA compared to six with CB [68].

PFA using the Farawave multielectrode pentaspline PFA catheter was compared to thermal ablation with either RF or CB in a 1:1 ratio in patients with paroxysmal AF in the ADVENT trial. ADVENT was a randomized, single-blind, noninferiority trial with 706 patients enrolled across 30 centers, making it the largest prospective trial in PFA to date [69]. Primary efficacy and safety endpoints were non-inferior. At 1 year, freedom from initial procedural failure, recurrent atrial tachyarrhythmia, antiarrhythmic drug use, or cardioversion was 73.3% in the PFA arm compared to 71.3% in the thermal ablation arm.

SPHERE Per-AF was a randomized, single-blind, non-inferiority trial of 420 patients with persistent AF undergoing ablation with a large-tip catheter with dual PF and RF energies (Sphere-9, Medtronic, Inc., Watertown, MA, USA) versus conventional RF ablation. The primary effectiveness endpoint was a composite of freedom from acute procedural failure, repeat ablation at any time, arrhythmia recurrence, and drug initiation or cardioversion after the 3-month blanking period. At 12 months, the primary effectiveness endpoint was observed for 73.8% and 65.8% in the investigational and control arms, respectively (*p* < 0.0001 for non-inferiority). Serious procedure-related or device-related events were also non-inferior between the groups, occurring in three and two patients in the investigational and control groups, respectively [70].

PULSE-EU was a first-in-human study of a multielectrode 30 mm spherical array catheter containing 122 gold-plated electrodes (Globe, Kardium Inc., Burnaby, BC, Canada). A total of 21 patients were assigned to either single PFA application per PV or three PFA applications per PV. PVI was acutely successful in 100% of patients in both groups; however, PV remapping at 2–3 month follow-up revealed durable PVI in only 62.5% of the single PFA application group compared to 100% in those receiving three PFA applications. There were no major adverse events reported; however, silent cerebral lesions (SCLs) were present in 18.7% of patients on screening brain MRI [75]. Follow-up data at 12 months demonstrated freedom from atrial arrhythmia of 84.2% and 80.0% in paroxysmal and persistent AF, respectively [71].

The FARADISE prospective global registry is a database for real-world clinical outcomes of patients undergoing AF ablation with the Farawave catheter (Boston Scientific Inc., Menlo Park, CA, USA). In 986 patients, 98.6% of PVs were isolated. Long-term efficacy data are not presently available. Acute device- or procedure-related serious adverse events occurred in 3.07% of cases [72].

The largest collection of real-world safety data comes from the MANIFEST-17K registry of 17,642 patients that underwent PVI with the Farapulse PFA system. Remarkably, there were no complications of atrioesophageal fistula, pulmonary vein stenosis, or persistent phrenic nerve palsy. There were five (0.03%) deaths. Other complications included tamponade (0.36%), stroke (0.12%), coronary spasm (0.14%), and vascular complications requiring intervention (0.30%) [73]. The catheters used in the clinical studies discussed are referenced in Supplementary Figure S1.

## 6. Discussion

PFA has emerged as a promising new technology for the treatment of cardiac arrhythmias, particularly AF. In comparison to thermal ablation techniques such as RF and cryoablation, PFA offers several important advantages.

IRE allows for targeted tissue-specific ablation of the myocardium while sparing vital neighboring structures such as the coronary arteries, phrenic nerve, and esophagus. The safety of PFA in preventing severe complications has been demonstrated in a real-world patient population in the MANIFEST-PF registry [73]. In over 17,000 patients there were no episodes of atrioesophageal fistula, PV stenosis, or persistent phrenic nerve paralysis. In the initial MANIFEST-PF registry data, there was a single case of phrenic nerve palsy persisting beyond 1 year [63].

PFA has been shown to be faster than thermal ablation in terms of left atrial dwell time and total procedural time. This has important implications for improved clinical efficiency, hospital resource utilization, and reduction in ablation wait times for patients. The rapid delivery of PFA to a vein can be completed within seconds in contrast to the longer duration required for point-by-point lesion delivery in RF. In the ADVENT trial, the mean procedural time was 105.8 min and 123.1 min in the PFA and thermal arms, respectively, corresponding to an average reduction in procedural time of 17.3 min. Left atrial dwell time was 59.4 min in the PFA arm compared to 83.7 min in the thermal arm [69].

In terms of the disadvantages of PFA, coronary spasm appears to be a unique complication and was noted in 25 patients (0.14%) in the MANIFEST-17K registry [73]. Higuchi et al. demonstrated that direct epicardial delivery of PFA to the coronary arteries resulted in acute spasm and chronic mild stenosis via neointimal hyperplasia [76]. This raises the question of the possible long-term risks of PFA on coronary artery injury/stenosis. In a comparative analysis of PFA and RF, PV narrowing or stenosis occurred in 12.0% and 32.5% of RF ablations, respectively, while none occurred with PFA [77]. Other complications such as vascular injury, cardiac perforation causing tamponade, and stroke appear to be less associated with PFA technology specifically and more related to catheter access and exchange, transseptal puncture, anticoagulation management, and catheter manipulation.

While periprocedural stroke is a recognized complication of catheter ablation, SCLs may go undetected and are of unclear prognostic significance [78]. PFA has been recognized to cause microbubbles during ablation visualized on intracardiac ultrasound (ICE), which may cause SCLs. In the inspIRE trial, SCLs were detected on MRI in four of the first six subjects. Following this, procedural adjustments were made, including implementing a 10 s pause between PFA applications, minimizing catheter exchanges, and stricter adherence to anticoagulation parameters. After these changes, only four further SCLs were detected in the remaining 33 patients. All lesions were resolved by 3 months [65]. PFA has also been associated with intravascular hemolysis, which in rare cases can be severe enough to trigger acute kidney injury. Venier et al. showed a significant inverse correlation between plasma haptoglobin levels and total number of PFA ablation lesions in a 68-patient study [42].

While early results are promising, long-term data on the efficacy and safety of PFA are still limited. Most studies to date have focused on short- to mid-term outcomes, and further studies are needed to confirm the durability of PFA lesions and the long-term benefits and risks. In a meta-analysis comparing PFA to thermal ablation, Gong et al. demonstrated that there was no significant difference in the freedom from atrial arrhythmia recurrence and the incidence of periprocedural complications [79]. The precise mechanisms of action and the factors influencing lesion formation in PFA still need to be fully understood. Variability in tissue responses and the potential for incomplete or non-durable lesions remain areas of active investigation. More research is needed to optimize parameters and improve consistency in outcomes. While PFA shows promise for AF ablation, its efficacy for other cardiac arrhythmias remains to be thoroughly investigated. Traditional thermal ablation methods have established roles in various arrhythmias, and whether PFA can be as versatile is unclear.

There are limited data on the use of PFA in the treatment of ventricular arrhythmias. While several small pre-clinical animal studies have demonstrated acute safety and feasibility, in-human studies are limited to case reports/series [80,81]. Atrial and ventricular myocardium exhibit important structural and functional differences that have significant relevance for ablation. The atrial myocardium is thinner and contains more gap junctions to facilitate rapid conduction. In contrast, ventricular myocardium is thicker with larger and more contractile proteins to accommodate higher pressures. The greater wall thickness of ventricular myocardium poses challenges to endocardial ablation of mid-myocardial, septal, and epicardial substrate with RF due to inability to achieve transmural ablation lesions. Thermal complications such as steam pops and char/coagulum are another limitation. Finally, risk of damage to coronary arteries in high-risk anatomical locations such as LV summit is a concern with RF. In a swine infarct model comparing PFA to RF ablation of LV scar, PFA produced uniform and well-demarcated lesions extending into deep layers of scar. In contrast, RF was limited to the subendocardium and was unable to penetrate layers of collagen and fat to reach deeper areas of scar [55]. Coronary vascular damage was seen with RF but not with PFA. These findings suggest that PFA may allow for more effective ablation of deep ventricular substrate and in anatomical locations at high-risk of damage to vital collateral structures while avoiding thermal complications. Improved catheter designs and optimized pulse parameters will be important in the ongoing development of PFA for VT ablation. Clinical studies comparing PFA to RF are required to gain a better understanding of PFA’s potential role in VT ablation.

Further research, particularly in vivo experiments or human tissue-based models, is needed to better understand the biophysical principles of human cardiomyocytes as it pertains to electroporation. Kaminska et al. utilize H9C2 cells, a rat myoblast line derived from neonatal heart tissue, as a model for cardiomyocytes [35]. While these cells share some similarities with mature cardiomyocytes, they lack full differentiation and the structural and functional characteristics of adult heart cells. This limits the direct applicability of the findings to human cardiac tissue.

## 7. Conclusions

In summary, this review provides a focused overview of electroporation biophysics as it pertains to PFA for the ablation of cardiac arrhythmias. Preclinical and clinical studies are discussed, which have advanced our understanding of how PFA can be harnessed to optimize safety and efficacy endpoints. PFA has several compelling advantages over traditional thermal ablation techniques, including selective tissue targeting, reduced procedure times, favorable safety profiles, and avoidance of thermal damage. The initial clinical experience with PFA is promising; however, long-term safety and efficacy data are required. Broader applicability with respect to the role of PFA in VT ablation and other focal arrhythmias in an area of ongoing research. As with any emerging technology, cautious optimism and rigorous clinical validation will be essential to fully realize the potential benefits of PFA while ensuring patient safety and treatment efficacy.

## Figures and Tables

**Table 1:** Pulsed field ablation clinical studies.

**Study, Year**	**Catheter (Company)**	**Methods**	**Efficacy Outcome**	**Safety Outcome**
Reddy et al., 2018 [56]	Endocardial: Basket/Flower Pentaspline, 12F (Iowa Approach Inc., Menlo Park, CA, USA) Epicardial: Linear cinching (Iowa Approach Inc.)	NRT *n* = 22 (15 endocardial, 7 epicardial) PAF Acute F/U	100% acute PVI (endocardial) 86% acute PVI (epicardial)	No adverse events
Reddy et al., 2020 [57]	Lattice-tip, 7.5F (Sphere-9, Affera Inc., Watertown, MA, USA)	NRT *n* = 76 PAF or PersAF 12-month F/U	100% acute PVI	No adverse events
IMPULSE, 2020 [58,59]	Basket/Flower Pentaspline, 12F (Farawave, Farapulse Inc., Newton, MA, USA)	NRT *n* = 40 PAF Acute and 12-month F/U	Combined analysis (total *n* = 121): 100% acute PVI 96% PV durability 85% free of atrial arrhythmia at 12-months	Combined analysis (total *n* = 121): 1 cardiac tamponade 1 pericardial effusion 1 vascular hematoma 1 TIA
PEFCAT, 2020 [58,59]	Basket/Flower Pentaspline, 12F (Farawave, Farapulse Inc.)	NRT *n* = 71 PAF Acute and 12-month F/U		
PEFCAT II, 2020 [59]	PVI: Basket/Flower Pentaspline, 12F (Farawave, Farapulse Inc.) CTI: Deflectable tetraspline, 12F (Faraflex, Farapulse Inc.)	NRT *n* = 10 PAF Acute and 12-month F/U		
Loh et al., 2020 [60]	Circular 14-polar, 8F (not specified)	NRT *n* = 10 PAF or PersAF Acute F/U	100% acute PVI	No adverse events
PersAFOne, 2020 [61]	PVI/LAPW: Basket/Flower Pentaspline, 12F (Farawave, Farapulse Inc.) CTI: Deflectable tetraspline, 12F (Faraflex, Farapulse Inc.)	NRT *n* = 25 PersAF Acute and 2–3 month F/U	100% acute PVI and LAPW ablation. 100% successful CTI (*n* = 13) Durability at 2–3 months: 96% PVI and 100% LAPW	No adverse events
PULSED AF, 2022 [62]	Circular lasso-type 9-electrode, 9F (PulseSelect, Medtronic, Inc., St. Brampton, ON, USA)	NRT *n* = 300 PAF or PersAF Acute and 12-month F/U	100% acute PVI Freedom from atrial arrhythmia at 12 months: 69.5% (paroxysmal), 62.3% (persistent)	1 stroke 1 cardiac tamponade 2 non-procedure related deaths during the F/U period
MANIFEST-PF, 2022 [63]	Basket/Flower Pentaspline, 12F (Farawave, Farapulse Inc.)	Retrospective registry *n* = 1568 PAF or PersAF Acute and 12-month F/U	99.2% acute PVI Freedom from atrial arrhythmia at 12 months: 81.6% (paroxysmal), 71.5% (persistent)	Procedure-related adverse events: 1.9% (*n* = 30) 1 death, 18 tamponade, 6 stroke, 2 vascular complications, 2 coronary artery spasm, 1 persistent phrenic nerve palsy
Reddy et al., 2023 [64]	Lattice-tip, bidirectional deflectable, 8F (Sphere-9, Medtronic, Inc.)	NRT *n* = 178 PAF or PersAF Acute and 12-month F/U	100% acute PVI, LA roof, mitral, and CTI. Freedom from atrial arrhythmia at 12 months: 78.3% (paroxysmal), 77.9% (persistent)	1 pericardial effusion
inspIRE, 2023 [65]	Variable-loop circular 10-electrode, 8.5F (Varipulse, Biosense Webster, Inc.)	NRT *n* = 226 PAF Acute and 12-month F/U	100% acute PVI Freedom from atrial arrhythmia at 12 months: 78.9%	No adverse events 8 SCLs.
admIRE, 2023 [66]	Variable-loop circular 10-electrode, 8.5F (Varipulse, Biosense Webster, Inc.)	NRT *n* = 277 PAF Acute and 12-month F/U	100% acute PVI Freedom from atrial arrhythmia at 12 months: 76.2%	Procedure-related adverse events: 2.9% (*n* = 8) including 3 cardiac tamponade
Urbanek et al., 2023 [67]	PFA: Basket/Flower Pentaspline, 12F (Farawave, Boston Scientific Inc.) CB: 28-mm (Arctic Front Advance, Medtronic, Inc.)	NRT *n* = 400 (200 PFA, 200 CB) PAF or PersAF Acute and 12-month F/U	PFA: 100% acute PVI, freedom from atrial arrhythmia at 12 months: 74.5% CB: 98% acute PVI, freedom from atrial arrhythmia at 12 months: 78.1%	PFA: 5 vascular access complication, 1 tamponade. CB: 7 vascular access complication, 3 persistent phrenic nerve palsy, 1 stroke/TIA, 1 esophageal injury, 1 hemoptysis
Schipper et al., 2023 [68]	PFA: Basket/Flower Pentaspline, 12F (Farawave, Boston Scientific Inc., Marlborough, MA, USA) CB: 31 mm (POLARx, Boston Scientific Inc.)	NRT *n* = 108 (54 PFA, 54 CB) PAF or PersAF Acute and 273 ± 129 day F/U	PFA: 100% acute PVI, 74% arrhythmia free survival CB: 99.5% acute PVI, 72% arrhythmia free survival	PFA: 2 tamponade CB: 2 vascular access complication, 1 TIA, 2 phrenic nerve injury
ADVENT, 2023 [69]	Basket/Flower Pentaspline, 12F (Farawave, Boston Scientific Inc.)	RCT, non-inferiority *n* = 706, 1:1 randomization to PFA vs. conventional thermal ablation (RF or cryoballoon) Acute and 12-month F/U	99.6% acute PVI (PFA) and 99.8% acute PVI (Thermal) Primary outcome at 12-months (freedom from composite of PVI procedural failure, recurrent atrial arrhythmia, AAD use and repeat ablation): PFA 73.3% vs. Thermal 71.3%, posterior probability of non-inferiority >0.999	Serious adverse events: PFA 2.1% (n = 6) vs. thermal 1.5% (*n* = 4), posterior probability of non-inferiority >0.999 PFA: 1 death, 2 tamponade or perforation, 1 TIA, 1 pericarditis, 1 pulmonary edema, 1 vascular access complication Thermal: 1 stroke, 1 pulmonary edema, 2 vascular access complication
SPHERE Per-AF, 2024 [70]	Lattice-tip, bidirectional deflectable, 8F (Sphere-9, Medtronic, Inc.)	RCT, non-inferiority *n* = 420, 1:1 randomization PFA/RF vs. RF alone 12-month F/U	Freedom from atrial arrhythmia at 12 months: 76.7% (PFA/RF) vs. 72.8% (RF), *p* < 0.0001 for non-inferiority	Serious adverse events: PFA/RF 1.4% (*n* = 3) vs. RF 1.0% (*n* = 2), *p* < 0.0001 for non-inferiority. PFA/RF: 1 pulmonary edema, 1 COPD exacerbation, 1 hemoptysis RF: 2 pulmonary edema
PULSE-EU, 2024 [71]	30 mm spherical array 122-electrode, 16F (Globe, Kardium Inc., Burnaby, BC, Canada)	NRT *n* = 48 PAF or PersAF Acute and 12-month F/U	100% acute PVI and posterior wall, 91% mitral isthmus 93.5% PVI durability at 3 months. Freedom from atrial arrhythmia at 12 months: 84.2% (paroxysmal), 80.0% (persistent)	1 pericarditis
FARADISE, 2024 [72]	Basket/Flower Pentaspline, 12F (Farawave, Boston Scientific Inc.)	Prospective global registry *n* = 986 PAF or PersAF Acute F/U	98.6% acute PVI	Procedure-related adverse events: 3.07% (*n* = 32) 1 vascular complication, 1 air embolism, 1 stroke, 1 hemolysis, 2 pericarditis, 2 pericardial effusion, 1 tamponade
MANIFEST-17K, 2024 [73]	Basket/Flower Pentaspline, 12F (Farawave, Boston Scientific Inc.)	Retrospective observational *n* = 17,642 PAF or PersAF Safety outcomes	Efficacy data not reported	Major adverse events 173 (0.98%), death 0.03%, stroke 0.12%, esophageal fistula 0%, PV stenosis 0%, phrenic nerve injury 0%, tamponade 0.36%, vascular complication with intervention 0.30%, coronary artery spasm 0.14%

AAD = antiarrhythmic drug, AF = atrial fibrillation, COPD = chronic obstructive pulmonary disease, CB = cryoballoon, CTI = cavotricuspid isthmus, F/U = follow-up, LA = left atrium, LAPW = left atrial posterior wall, *n* = number, NRT = non-randomized trial, PAF = paroxysmal atrial fibrillation, PersAF = persistent atrial fibrillation, PFA = pulsed field ablation, PV = pulmonary vein, PVI = pulmonary vein isolation RF = radiofrequency ablation, SCL = silent cerebral lesion, TIA = transient ischemic attack.

## Supplementary Materials

The following supporting information can be downloaded at: https://www.mdpi.com/article/10.3390/bioengineering12040329/s1, Figure S1: Schematic illustration of PFA catheters referenced to the clinical studies.
