# Supplementary material for: Pulsed Field Ablation: A Review of Preclinical and Clinical Studies
Source: Bioengineering (Basel). 2025 Mar 22;12(4):329. doi: 10.3390/bioengineering12040329 (PMC12024434; doi:10.3390/bioengineering12040329)

Supplementary Files

Supplementary Figure S1: Schematic illustration of PFA catheters referenced to the clinical studies. (Iyengar 2023 [46] / CC BY 4.0, reproduced and modified with permission).

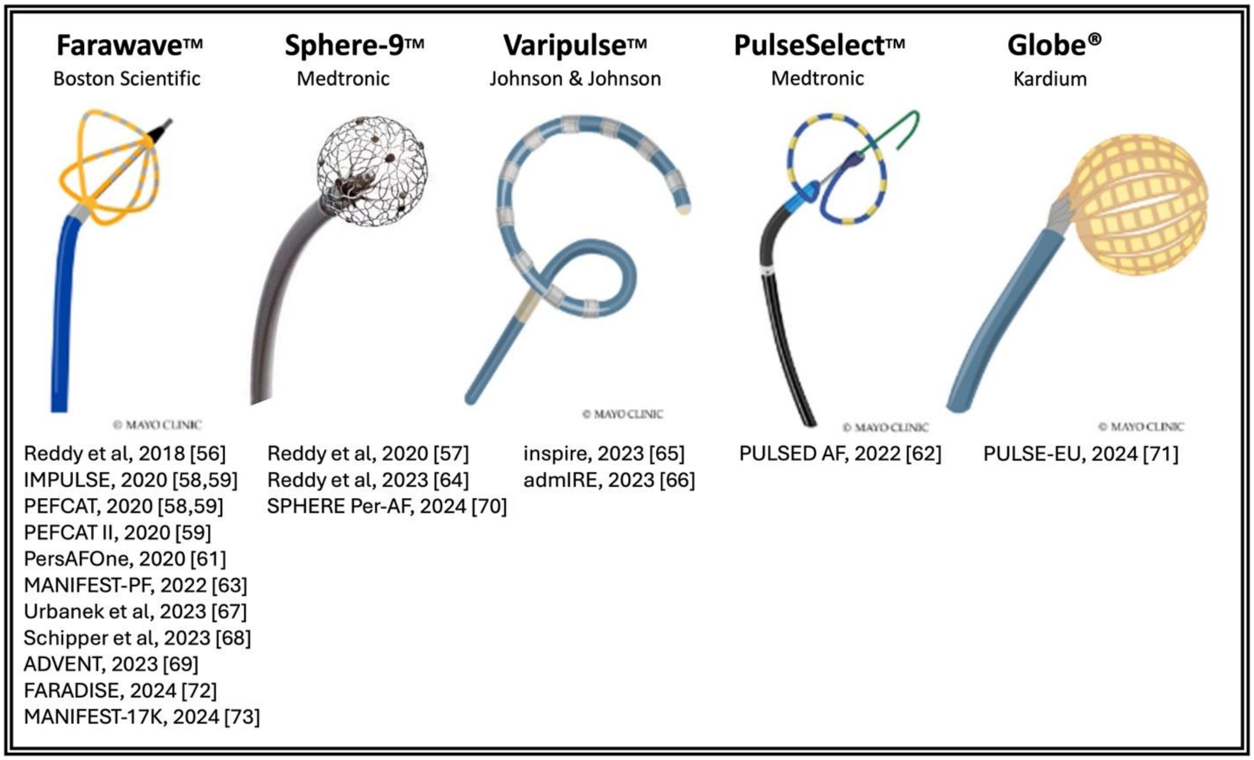

Supplement: Supplementary file 1 [file bioengineering-12-00329-s001.zip › bioengineering-3370650-supplementary.pdf]
